# Morphological changes in the subthalamic nucleus of people with mild-to-moderate Parkinson’s disease: a 7T MRI study

**DOI:** 10.1038/s41598-020-65752-0

**Published:** 2020-05-29

**Authors:** Rémi Patriat, Jacob Niederer, Jordan Kaplan, Sommer Amundsen Huffmaster, Matthew Petrucci, Lynn Eberly, Noam Harel, Colum MacKinnon

**Affiliations:** 1 Center for Magnetic Resonance Research, Department of Radiology, University of Minnesota, Minneapolis, MN USA; 2 Department of Neurology, University of Minnesota, Minneapolis, MN USA; 3 Division of Biostatistics, School of Public Health, University of Minnesota, Minneapolis, MN USA

**Keywords:** Nervous system, Brain imaging, Magnetic resonance imaging, Parkinson's disease

## Abstract

This project investigated whether structural changes are present in the subthalamic nucleus (STN) of people with mild-to-moderate severity of Parkinson’s disease (PD). Within-subject measures of STN volume and fractional anisotropy (FA) were derived from high-resolution 7Tesla magnetic resonance imaging (MRI) for 29 subjects with mild-to-moderate PD (median disease duration = 2.3±1.9 years) and 18 healthy matched controls. Manual segmentation of the STN was performed on 0.4 mm in-plane resolution images. FA maps were generated and FA values were averaged over the left and right STN separately for each subject. Motor sign severity was assessed using the Movement Disorders Society Unified Parkinson’s Disease Rating Scale (MDS-UPDRS). Linear effects models showed that STN volume was significantly smaller in the PD subjects compared to controls (p = 0.01). Further, after controlling for differences in STN volumes within or between groups, the PD group had lower FA values in the STN compared to controls (corrected p ≤ 0.008). These findings demonstrate that morphological changes occur in the STN, which likely impact the function of the hyperdirect and indirect pathways of the basal ganglia and movement control.

## Introduction

The subthalamic nucleus (STN) is a critical hub of both the indirect (putamen - globus pallidus externus - STN - globus pallidus internus) and hyperdirect (cortex-STN) pathways of the basal ganglia. Changes in the excitability and firing patterns of the STN following the degeneration of nigrostriatal dopaminergic neurons are considered to be central to the expression of many of the motor and non-motor signs of PD^1^. Yet, it is now increasingly appreciated that these pathological changes in the firing patterns of neurons arise not only from alterations in the excitability of striatal output pathways, but are also mediated by changes in the morphology and function of synaptic connections across the basal ganglia network^2,3^. Evidence from experiments in animal models of parkinsonism have shown there is a profound loss of cortico-subthalamic nucleus (STN) inputs (hyperdirect pathway) and marked heterosynaptic changes in the connectivity between the STN and globus pallidus externus following degeneration of midbrain dopaminergic neurons^4,5^. Moreover, these changes occur early after nigrostriatal dopaminergic denervation and may play a pivotal role in the expression of motor signs^5^.These animal models do not recapitulate the long prodromal period of degeneration in idiopathic PD, however, if comparable changes occur in humans with PD, then alterations in magnetic resonance imaging (MRI) measures of morphology and microstructure of the STN would be predicted in relatively early stage of disease.

Resting state functional MRI studies in humans have provided evidence that there are changes in the function of cortico-subthalamic pathways in people with early stage, untreated PD^6,7^. Yet, to date, there has been little *in vivo* evidence of morphological or microstructural changes in extranigral basal ganglia structures, including the STN, in humans with PD. One previous MR study (at 3 Tesla) reported a smaller (average of 48%) volume of the STN in a small cohort of individuals with advanced disease (mean 10.8 years post-diagnosis)^8^, while others found no differences^9^. This lack of consensus likely arises from errors in the estimation of the STN borders due to the relatively low spatial resolution and signal-to-noise ratio of the scanners used (1.5 or 3T) (often resulting in fewer than 10 large voxels to depict the structure) and the use of “one-size-fit-all” anatomical templates which are inadequate for modelling small, geometrically complex structures, such as the STN. A recent study at 7T has shown that there is considerable variance in the shape, size and orientation of the nucleus across individuals^10^. For this reason, it is essential that estimates of the volume of the STN be derived from precise segmentation of the borders of the nucleus within-individuals.

The goal of this study was to use ultra-high-field MRI to examine whether morphological changes can be seen in the STN of people with mild-to-moderate severity of PD. Ultra-high-field (7T) MRI was used to precisely view the boundaries of the STN^10–12^, thus enabling analysis at the individual level^10,13^. Specifically, we tested the hypotheses that the volume and fractional anisotropy (a measure of tissue microstructure) of the STN is reduced in people with mild-to-moderate motor severity of PD compared with matched control subjects.

## Results

### Participants

There was no significant difference in the number of male and female participants (PD: 12F and 17M, controls: 9F and 9M, χ^2^ = 0.076, df =1, p = 0.78) or age (PD = 63.5 ± 7.6 years; healthy control group = 61.2 ± 8.3 years) (t = 0.96, df = 32.71, p = 0.35). There was also no significant difference in whole brain volume between group (Control: 1058 ± 95cm^3^; PD: 1076 ± 91cm^3^; p = 0.75).

### STN volume

**Figure 1:**
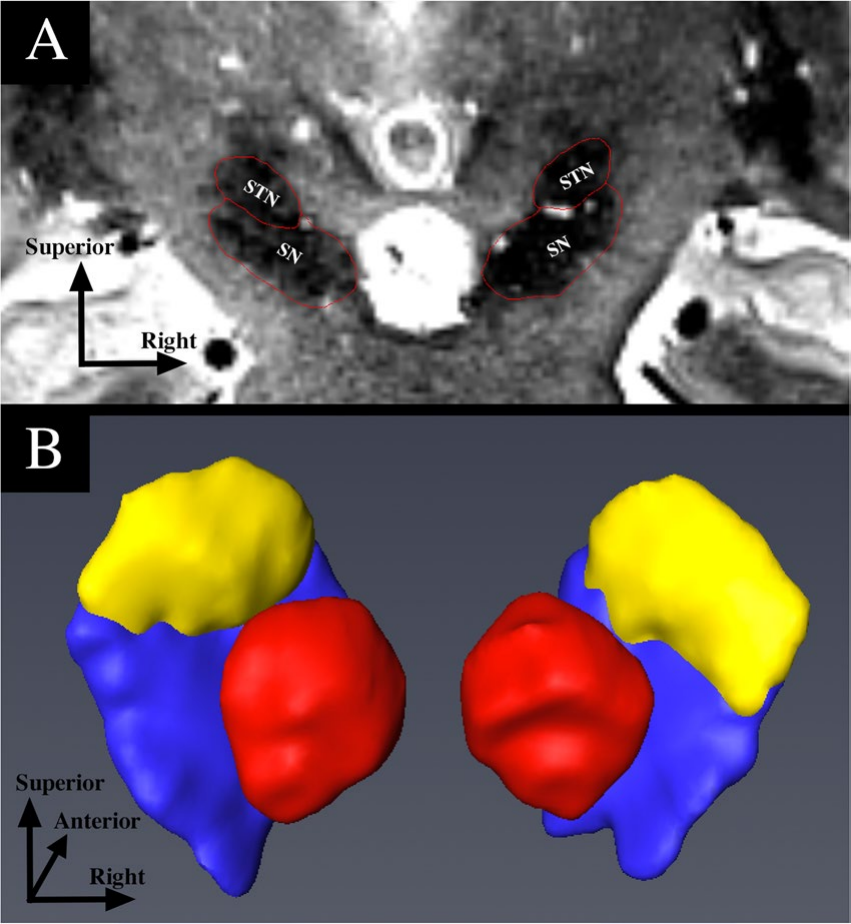
Example of STN segmentation and 3D reconstruction for one patient in the coronal orientation. (**A**) Identification of the STN on a T2 MRI slice. (**B**) 3D rendering of the STN, SN and RN. Yellow = STN. Blue = SN. Red = RN. STN = subthalamic nucleus. SN = substantia nigra. RN = red nucleus.

**Figure 2:**
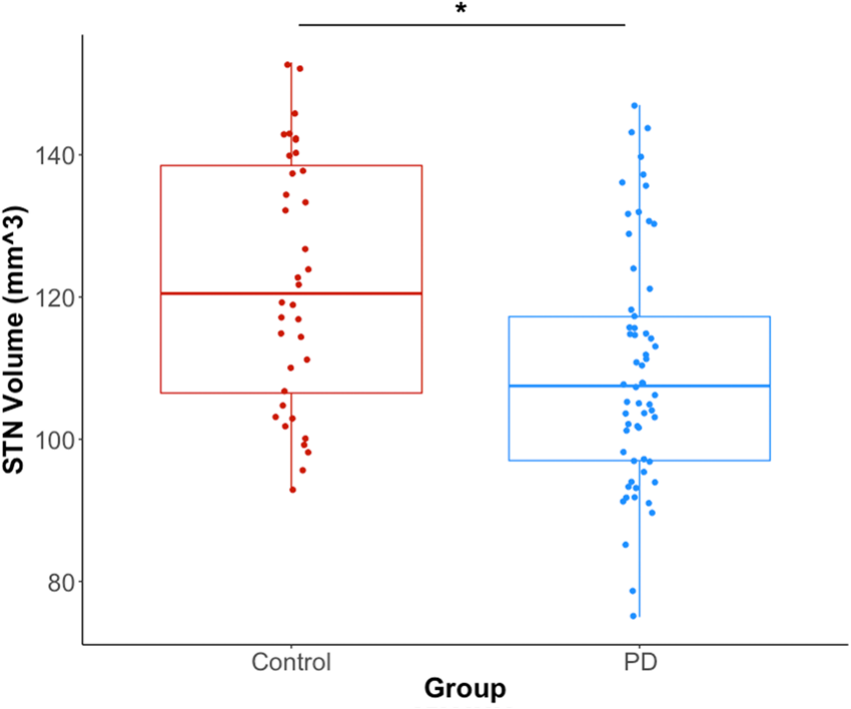
STN volume differences between the two groups. Blue indicates data for the PD group. Orange indicates data for the healthy control group. EPD = early Parkinson’s disease group. STN = subthalamic nucleus. * = Statistical significance p < 0.05 from a linear mixed model effect. The 25^th^ and 75^th^ percentiles are represented by the lower and upper boundaries of each box with median values represented by the middle band within the box.

Figure 1 shows an example of an STN segmentation. The mean *raw* STN volume was 122 ± 18mm^3^ for the healthy control subjects (right volume = 124 ± 19mm^3^; left volume = 121 ± 17mm^3^) and 110 ± 17mm^3^ for the PD group (right volume = 110 ± 18mm^3^; left volume = 111 ± 16mm^3^) (Fig. 2).

In the first linear effects model, both healthy control and PD groups were included to model *normalized* STN volume. The *normalized* STN volumes were 116 ± 13 mm^3^ for the controls and 103 ± 17 mm^3^ for the PD group. Age, sex, side and STN FA were considered as adjusting variables, but their effects were not statistically significant (p > 0.10) and were removed from the model. The effects of group were statistically significant and were retained in the model. The model estimated difference in *normalized* STN volume between healthy control and PD groups was found to be 12mm^3^ (95% confidence interval^3,21^, t = −2.67, df = 45, p = 0.01, regression coefficient = −12.3), indicating an 11.2% smaller average *normalized* STN volume in the PD group compared to the healthy controls.

In the second linear effects model, factors contributing to the *normalized* STN volume in the PD group were examined. Age, sex, side, and STN FA were considered as adjusting variables, but their effects were not statistically significant (p > 0.077). There was an inverse relationship between clinical measures of motor severity (total MDS-UPDRS III score) and *normalized* STN volume (i.e. participants with increased motor severity had decreased STN Volume) but this relationship across PD participants did not reach statistical significance (p = 0.056). Lateralized MDS-UPDRS III scores were used in a separate model but the results were not statistically significant (p = 0.13).

### STN FA

**Figure 3:**
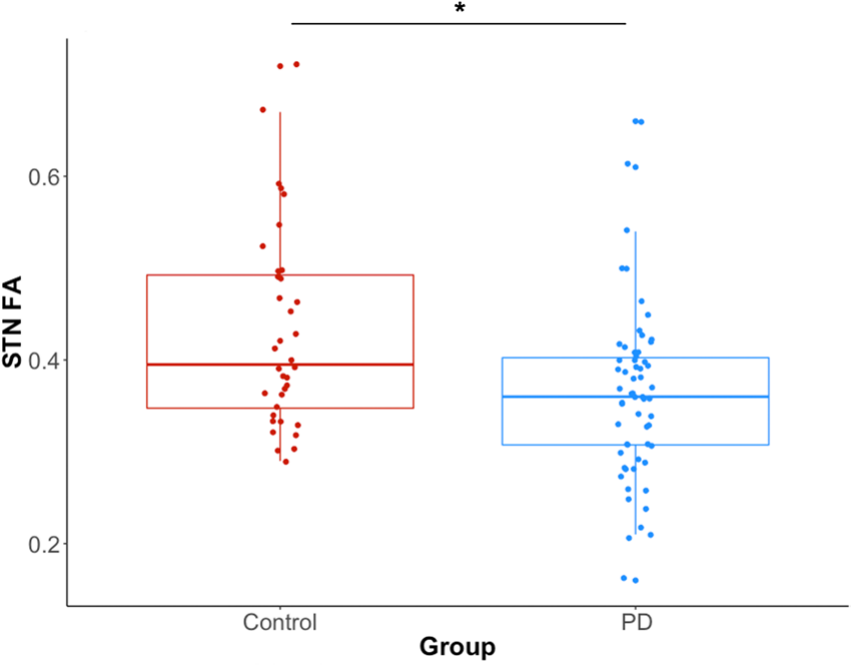
STN-FA differences between the two groups. Blue indicates data for the PD group. Orange indicates data for the healthy control group. FA = fractional anisotropy. EPD = early Parkinson’s disease group. STN = subthalamic nucleus. * = Statistical significance p < 0.05 from a linear mixed model effect. The 25^th^ and 75^th^ percentiles are represented by the lower and upper boundaries of each box with median values represented by the middle band within the box.

Mean STN FA values were 0.36 ± 0.09 for the PD group and 0.43 ± 0.11 for the healthy control group (Fig. 3). No significant difference in STN FA was observed between the left and right sides for both groups (p = 0.50). Age, sex, side and *normalized* STN volume were considered as adjusting variables, but their effects were not statistically significant (p > 0.08). Only the effect of subject group was statistically significant. The model estimated difference in STN FA between the healthy control and PD groups was found to be 0.07 (95% confidence interval [0.02, 0.12], t = −2.76, df = 45, p < 0.008, regression coefficient = −0.07), indicating a 19% higher average FA in the control subjects compared with the PD group.

In the second statistical model, factors contributing STN FA in the PD group were examined. Adjusting factors of age, sex and side were not significant. The factor of MDS-UPDRS III score was also not significant (p = 0.10). The adjusting factor of STN volume was statistically significant (t = −2.67, df = 28 *p* = 0.01, regression coefficient = −0.002). An inverse association between *normalized* STN volume and STN FA was observed. Lateralized MDS-UPDRS III scores were used in a separate model but the results were not statistically significant (p = 0.41).

## Discussion

This study is the first to show that people with mild-to-moderate PD show significant changes in STN morphology (volume) and microstructural organization (FA) compared with healthy older adults. These findings are consistent with the idea that neurodegenerative changes in the basal ganglia of people with PD are not confined to the substantia nigra pars compacta, and include alterations in the morphology and microstructure of extranigral structures in the basal ganglia-thalamo-cortical loop.

Recent work in both rodent and non-human primate models of parkinsonism have provided evidence that the degeneration of midbrain dopaminergic neurons is associated with the loss of hyperdirect pathway input, including a reduction in axo-dendritic and axo-spinous synapses and subsequent attenuation of cortico-STN transmission^4,5^. Alterations in hyperdirect pathway input to the STN were further accompanied by heterosynaptic increases in the strength and number of indirect pathway (globus pallidus externus) inputs to the STN^14,15^. The development of synaptic ultrastructural and functional changes in the STN following acute lesions of midbrain dopaminergic neurons precedes the emergence of parkinsonian motor signs by days to weeks^16,17^, suggesting that plastic changes in the hyperdirect and indirect pathways to the STN contribute to the early expression of motor impairment. However, these animal models of parkinsonism do not recapitulate the pathophysiology and progression of PD, therefore the extent to which comparable ultrastructural and functional changes occur in humans is unclear.

A variety of studies using diffusion MR have provided evidence of significantly reduced FA in the substantia nigra of people with PD (for review see^18^). Reduced FA in the substantia nigra has been interpreted to reflect structural changes associated with degeneration of nigrostriatal dopaminergic neurons due to the relationship of these changes to disease severity. Our findings add to growing evidence of morphological changes in extranigral structures in people with mild-to-moderate severity of PD. Significant increases in mean diffusivity (also considered as an index of putative degeneration) in PD have been reported in the pallidum and putamen^18^, but the results of studies using measures of FA in extranigral basal ganglia nuclei have been equivocal^18^. Studies using diffusion tensor-based tractography have consistently demonstrated significant changes in projection (including corticospinal pathways, which would include cortico-STN collaterals), callosal and association fibers of sensorimotor and premotor cortices in people with mild-to-moderate PD compared with healthy older adults^19–21^. Paradoxically, the FA of these white matter pathways has been shown to be significantly increased relative to controls. Currently, the structural changes that mediate increases in FA of white matter are poorly understood. Early stage PD is also associated with reductions in frontal cortical grey matter, as assessed by measures of cortical thinning, particularly in those with cognitive impairment^22,23^. Based on deformation based morphometry measures obtained from the Parkinson’s Progression Markers Initiative data set, Zeighami *et al*. (2019a,b)^24,25^ recently described a network of subcortical and cortical atrophy in people with *de novo* (untreated) PD that included cortical areas, the lower brainstem and basal ganglia. Taken together, these findings suggest that morphological changes occur in extranigral nuclei, white matter pathways and cortical grey matter in people in the mild-to-moderate PD.

In the present study, the volume of the right and left STN of the PD subjects were smaller by an average of 12 mm^3^ and 11% relative to the control subjects. By way of comparison, changes in the volume of the substantia nigra in the order of 11–22% have been reported in people with PD relative to healthy controls^26^. The ultra-high field, high-resolution methods used in this paper allowed us to visualize the borders of the STN and thus reconstruct the 3D volume of the STN within-individuals without the use of a template or warping of the images to an atlas. Duchin *et al*.^10^ recently showed that there is considerable variability in the location, orientation and shape of the STN across individuals; thus, approaches that use a template and image warping are likely to yield considerable errors in estimates of volume. This is especially true for small structures, like the STN. Several previous studies that derived STN volumes from low-resolution data sets and/or template approaches have yielded values that were higher and lower than the values obtained in the present study^27–30^. However, the STN volumes obtained in this study were consistent (within two standard deviations) with those reported in other high-resolution 7T datasets^10,12,31^, 9.4T datasets^32^ as well as values reported from postmortem histology studies^33–37^. The STN volume differences between the PD and control groups were also consistent with a previous report that tested individuals with more advanced disease but similar age^8^. It is noteworthy that there was a considerable range in both STN volumes and clinical ratings of motor disease severity across individuals with PD. Within the PD group, there was an inverse relationship between STN volumes and MDS-UPDRS part III motor scores, such that the participants with greatest motor severity having the lowest STN volumes, however this relationship did not reach statistical significance (p = 0.056). The morphological changes contributing to reduced STN volume are unknown at this time, but the fact that individuals with the smallest normalized STN volume presented with the highest severity of motor impairment suggests that these changes impact the function of basal ganglia motor pathways.

The second main finding from our study was the observation that the STN FA was significantly reduced in the PD group when compared with controls. On average, the STN FA in the PD group was reduced by 16%.Decreases in FA within the posterior region of the substantia nigra have been interpreted to reflect microstructural organization changes associated with nigrostrial dopaminergic degeneration^18^. In the MPTP model of parkinsonism, diffusion MR measures correlate with MPTP dose, nigral dopaminergic cell bodies count and the emergence of motor symptoms^38,39^. Although the relationships between white or grey matter integrity, fiber or cell count and diffusion MR measures, including FA, are poorly understood, the loss of cortico-STN input and proliferation of pallidal-STN synaptic connectivity is likely to result in alterations in anisotropy measures. Accordingly, our findings are consistent with the idea that changes in the ultrastructure and density of synaptic connections within the STN are present at a relatively early stage of disease.

Several potential confounds and limitations of the study are worthy of discussion. First, care was taken to minimize movement artifacts by using scan protocols that were optimized for the study of people with PD^10–13^. Second, ultra-high-field imaging, such as 7T, can be prone to increased distortions and signal loss when compared with 1.5T and 3T scanners, however, Duchin *et al*.^40^ have shown that differences at the level of the basal ganglia are negligible. Last, despite the fact that the median time since diagnosis in our PD study cohort was 1.4 years, there was considerable heterogeneity in the expression of motor symptoms. One half of the cohort had MDS-UPDRS III scores in the mild motor severity range (18–32) while the other half were assessed in the moderate range (33–58)^41^. The majority of the subjects were receiving oral dopamine replacement therapy and/or dopamine agonist. Since these agents could potentially modify the plasticity of basal ganglia thalamocortical pathways, it remains to be seen if similar findings would be observed in a *de novo* (untreated) cohort.

While degeneration of the substantia nigra pars compacta is the pathological hallmark of PD, it is now recognized that both degenerative and compensatory plastic changes occur in extranigral structures and pathways^2^. Here we show that the volume and fractional anisotropy (a measure of microstructure) of the STN are significantly reduced in people with mild-to-moderate motor severity of PD. These findings demonstrate that degenerative-like changes in the morphology and structural organization of the STN occur in people with PD and these alterations are related to the expression of motor signs.

## Methods

### Participants

Fifty participants completed testing for this study. Exclusion criteria included a significant neurological disorder other than PD, a Montreal Cognitive Assessment score of less than 22^42^, and standard MRI exclusion criteria. Two participants, one PD patient and one control, were excluded post-hoc from the analysis due to image quality issues (e.g. excessive subject motion). One more participant was excluded due to a change in diagnosis from PD to progressive supranuclear palsy. Severity of motor signs were assessed using the Movement Disorder Society-Unified Parkinson’s Disease Rating Scale Part III (MDS-UPDRS III) motor evaluation. All participants had mild-to-moderate severity of motor disease as defined by a score of between 0 to 58^43^. Disease stage was defined based on Hoehn and Yahr score. Demographic and levodopa equivalent dose data were also collected. The final data set included data from 29 individuals with mild-to-moderate severity of PD. Summary of the demographic and clinical characteristics is provided in (Table 1). Testing was conducted in the practically-defined off medication state following overnight withdrawal of levodopa and dopamine agonists and/or 48-hour withdrawal from long-acting dopaminergic medications. Age- and sex-matched controls were recruited from the community. The study was approved by the Institutional Review Board at the University of Minnesota and informed consent was obtained prior to inclusion into the study. Written informed consent was obtained from all participants. All experiments were performed in accordance with relevant guidelines and regulations.

**Table 1:** Demographics table. MoCA = Montreal Cognitive Assessment; MDS-UPDRS III = Movement Disorders Society Unified Parkinson’s Disease Rating Scale, Part III Motor Examination; LED = Levodopa Equivalent Dose.

Group	Sample Size	Sex	Age (years)	Disease Duration (median, years)	Hoehn & Yahr Stage	MDS-UPDRS III	MoCA	LED
PD	29	12F/17M	65.0 ± 7.6	1.5 ± 1.9	2.0 ± 0.4	31.0 ± 11.4	27.8 ± 1.7	300 ± 242
Controls	18	9F/9M	59.5 ± 8.3	N/A	N/A	N/A	26.8 ± 2.1	N/A

All values are median ± 1 standard deviation.

### Scanning protocol

Participants were scanned on a 7T MRI scanner (Magnetom 7T Siemens, Erlangen, Germany). The scanner was equipped with SC72 gradients capable of 70 mT/m and a 200 T/m/s slew rate using a 32-element head array coil (Nova Medical, Inc., Burlington, MA, USA). Whenever subject head size enabled enough space in the coil, dielectric pads were utilized in order to enhance signal in the temporal regions.

The scanning protocol consisted of: T2-weighted coronal slab centered on the region of the STN and oriented parallel to the brainstem (0.4 × 0.4 × 1.0 mm^3^, about 7 minutes), diffusion-weighted images (DWI) covering the whole brain (50 directions, b-value = 1500 s/mm^2^, 4 additional b0-volumes, 1.25 mm isotropic, 6.5 minutes) and a T1-weighted image (0.6 mm isotropic, 6.5 minutes). In order to better correct for distortion in the DWI dataset, these images were acquired twice, each with different phase encoding directions: anterior-posterior and posterior-anterior.

### Image processing & analysis

At 7T, the T2 images with 0.4 mm × 0.4 mm in-plane resolution showed the STN as a clear hypointense structure directly superior to the SN, inferior to the zona incerta, medial to the internal capsule, and lateral to the thalamic fasciculus in the coronal plane (Fig. 1). Manual segmentation of each STN was performed slice-by-slice in Avizo software (FEI, Hillsboro, OR, USA) by two expert raters until a consensus was reached. This generated a 3-dimensional model of the structure of interest. Note that the raters were blinded to the subject group by having the data presented to them for processing in a non-discriminate order and by using deidentified subject IDs. This protocol and process have been previously published^10,13^. The STN masks were brought to the same space as the subject’s diffusion image space by way of affine registration using ANTS. Each registration was verified visually.

DWI preprocessing steps included: motion, susceptibility, and Eddy current distortions correction using FSL’s eddy and topup algorithms. For each subject, FA maps were generated using FSL’s dtifit. FA values were averaged for the left and right STN separately and were computed in each subject’s native diffusion image space using AFNI’s 3dROIstats^44^. Finally, the T1 image was used to compute whole brain volume. The whole brain volume measure was used to normalize STN volumes for each subject in our statistical analyses to ensure that potential STN volume changes were not related to whole brain volume differences between groups.

### Statistical analysis

For all statistical tests performed, a p value of ≤ 0.05 was set *a priori* to indicate statistical significance. Values are expressed as mean ± SD unless otherwise stated. The open-source R software environment (version 3.5.2) and *nlme* were used to perform two linear mixed-effects models using the restricted maximum likelihood (REML) approach to examine the effects of independent factor and covariate variables on normalized STN volume and STN FA, separately. All models tested age, sex and side as potential adjusting variables. The first model included healthy control and early Parkinson’s disease groups (factor of group). The second model was exclusive to the early Parkinson’s disease group in order to examine the relationship between MDS-UPDRS part III motor score and normalized STN volume and STN FA, separately. Because normalized STN volume and STN FA values were measured on the right and left sides within each subject, the models controlled for within-subject variance by including random effects for subject. Additionally, normalized STN FA was included as a covariate of no interest when modeling normalized STN volumes and vice versa. For each model, non-significantly associated covariates were then dropped and the model retested to obtain the final statistics. Percent difference in the measures tested (e.g. *normalized* STN or STN FA) of the PD group compared to the control group was computed as the difference in the mean of the measure of interest (Mean_PD_ - Mean_controls_) times 100 divided by the mean for the control group (Mean_controls_).

## Data Availability

The data including normalized STN Volumes, FA values, MDS-UPDRS scores and limited demographics data are available upon request along with the R code, which used for all statistical analyses.
